# Vaginal Nugent Lactobacillus morphotype score and endometrial pathogen detection: a two-centre retrospective study

**DOI:** 10.3389/fendo.2026.1926579

**Published:** 2026-09-14

**Authors:** Kiyotaka Kawai, Shogo Nishii, Nanako Iwami, Hirobumi Kamiya

**Affiliations:** 1 Reproductive Medicine, Kameda IVF Clinic Makuhari, Chiba, Japan; 2 Reproductive Medicine, Kamiya Ladies Clinic, Sapporo, Japan

**Keywords:** assisted reproduction, chronic endometritis, endometrial dysbiosis, gram stain, non-invasive assessment, vaginal microbiota

## Abstract

**Introduction:**

This two-centre retrospective observational study investigated whether the vaginal Nugent *Lactobacillus* morphotype score (LMS) is associated with endometrial pathogen detection and intrauterine *Lactobacillus* abundance in women with infertility.

**Methods:**

The study included 282 women (Centre A, n = 92; Centre B, n = 190) who underwent simultaneous vaginal Nugent scoring and endometrial quantitative PCR between March and December 2025. Women who had used antibiotics within one month or any probiotics before testing were excluded. Associations were assessed by Spearman rank correlation and by multivariable logistic regression adjusted for centre, age, BMI, and recurrent implantation failure.

**Results:**

LMS was positively associated with endometrial pathogen detection (ρ = +0.229, p < 0.001). After adjustment for centre, age, BMI, and recurrent implantation failure, each one-point increase in LMS was associated with higher odds of pathogen detection (adjusted OR 1.40, 95% CI 1.17–1.68; p < 0.001). However, discriminatory performance was limited (AUC 0.636, 95% CI 0.567–0.705), and pathogen detection remained 23.3% at LMS 0. LMS was negatively correlated with total intrauterine *Lactobacillus* (ρ = −0.488, p < 0.001) and *L. crispatus* (ρ = −0.411, p < 0.001). All 56 women with LMS 4 were *L. crispatus*-negative; however, when LMS 4 was treated as a positive result, sensitivity for *L. crispatus* absence was only 30.8%.

**Discussion:**

Vaginal LMS is an associated preliminary non-invasive indicator, but it cannot substitute for direct endometrial assessment or reliably exclude endometrial pathogen detection.

## Introduction

The endometrial microbiota has been receiving increasing attention as a factor influencing reproductive outcomes. Although the uterine cavity was historically considered sterile, advances in next-generation sequencing (NGS) technology have demonstrated that the endometrium harbours a distinct microbial community (1–3). Mitchell et al. (4) detected vaginal-origin bacteria in 52 of 58 women (90%) undergoing hysterectomy, challenging the longstanding concept of a sterile uterine environment. Endometrial microbiota dysbiosis has been associated with infertility, recurrent implantation failure (RIF), and recurrent pregnancy loss (5–7). Furthermore, Moreno et al. reported that a relative abundance of *Lactobacillus* below 90% is associated with reduced implantation and live birth rates (2). These findings highlight the clinical importance of accurately assessing the endometrial microbiota in reproductive medicine.

Recent reviews have highlighted that microbial alterations are being investigated across a broad range of gynecological conditions, including endometrial disorders, while also emphasising the heterogeneity of sampling and analytical methods (8). In reproductive medicine, chronic endometritis illustrates the need for better-standardised diagnostic approaches integrating histology, microbial testing, and clinical context; however, universally accepted diagnostic and treatment pathways remain unavailable (9).

Despite growing recognition of its importance, the clinical utility of endometrial microbiota assessment remains uncertain, and a gap persists between research findings and clinical application (10). Importantly, recent evidence has challenged the assumption that modifying vaginal microbiota improves reproductive outcomes. In a randomised, double-blind, placebo-controlled trial of 338 *in vitro* fertilisation (IVF) patients, Haahr et al. demonstrated that treatment of vaginal dysbiosis with clindamycin and *Lactobacillus crispatus* (LACTIN-V) did not improve clinical pregnancy rates (11). Similarly, meta-analyses have shown that, while abnormal vaginal microbiota is associated with increased early pregnancy loss and lower clinical pregnancy rates, the effect of treatment is limited (12). These findings suggest that correcting vaginal dysbiosis alone does not necessarily improve reproductive outcomes. They provide a rationale for studying the endometrial microbial environment separately, while its causal and prognostic significance remains under investigation.

However, the diagnostic value of vaginal microbiota assessment may not be negligible. The vagina and endometrium are anatomically contiguous, and ascending migration of vaginal bacteria has been reported (13, 14). If a simple assessment of the vaginal microbiota is associated with selected endometrial microbial features, vaginal testing could provide preliminary non-invasive contextual information about the endometrial environment; however, association alone would not establish surrogate validity. The value of vaginal microbiota assessment may therefore lie not in guiding vaginal treatment, but in reflecting the state of the endometrial microbiota. Vaginal microbiome assessment is non-invasive and correlates with the upper reproductive tract (10), further supporting this indirect clinical value.

Hiratsuka et al. (7) have recently proposed a conceptual framework classifying chronic endometritis into (1): cases resolved by antibiotics alone, (2) cases requiring both antibiotics and microbiome restoration, and (3) cases where microbial dysbiosis is not the primary cause. This framework underscores the importance of individualised treatment rooted in combined endometrial assessment (CD138 immunohistochemistry and endometrial microbiota testing).

The Nugent score is the gold standard for diagnosing bacterial vaginosis (BV), semi-quantitatively evaluating three morphotypes using Gram staining: *Lactobacillus* morphotype (large gram-positive rods; LMS: 0–4), Gardnerella/Bacteroides morphotype (small gram-negative to gram-variable rods; GMS: 0–4), and curved gram-variable rods (Mobiluncus morphotype; MMS: 0–2); the total Nugent score ranges from 0–10 (15). The present study focused on the *Lactobacillus* morphotype score (LMS: 0–4) and examined its association with endometrial pathogen detection, while intrauterine *Lactobacillus* quantity was assessed as a secondary measure. This design allowed LMS to be evaluated as a candidate preliminary indicator of selected endometrial microbial features, without treating it as a substitute for direct endometrial assessment.

## Materials and methods

### Study design and participants

In this two-centre retrospective observational study, we identified 282 consecutive eligible women with infertility who underwent simultaneous vaginal Nugent scoring and endometrial qPCR analysis between March and December 2025 at two specialised infertility centres: Centre A (n = 92; approximately 1,100 oocyte retrieval cycles annually) and Centre B (n = 190; approximately 2,100 oocyte retrieval cycles annually).

The exclusion criteria were as follows: use of antibiotics within 1 month prior to testing and use of probiotics. The primary endpoint was the correlation between LMS and endometrial pathogen detection (Spearman rank correlation). The secondary endpoints included the association between LMS and intrauterine *Lactobacillus* total as well as that between LMS and *Lactobacillus* species (*L. crispatus*, *L. iners*, *L. gasseri*, and *L. jensenii*). All eligible women during the predefined study period were included, and no participant had missing data for the primary exposure, primary outcome, or prespecified covariates. Participant identification and exclusions are summarised in **Figure 1**. This study was reported in accordance with the Strengthening the Reporting of Observational Studies in Epidemiology (STROBE) guideline.

**Figure 1:**
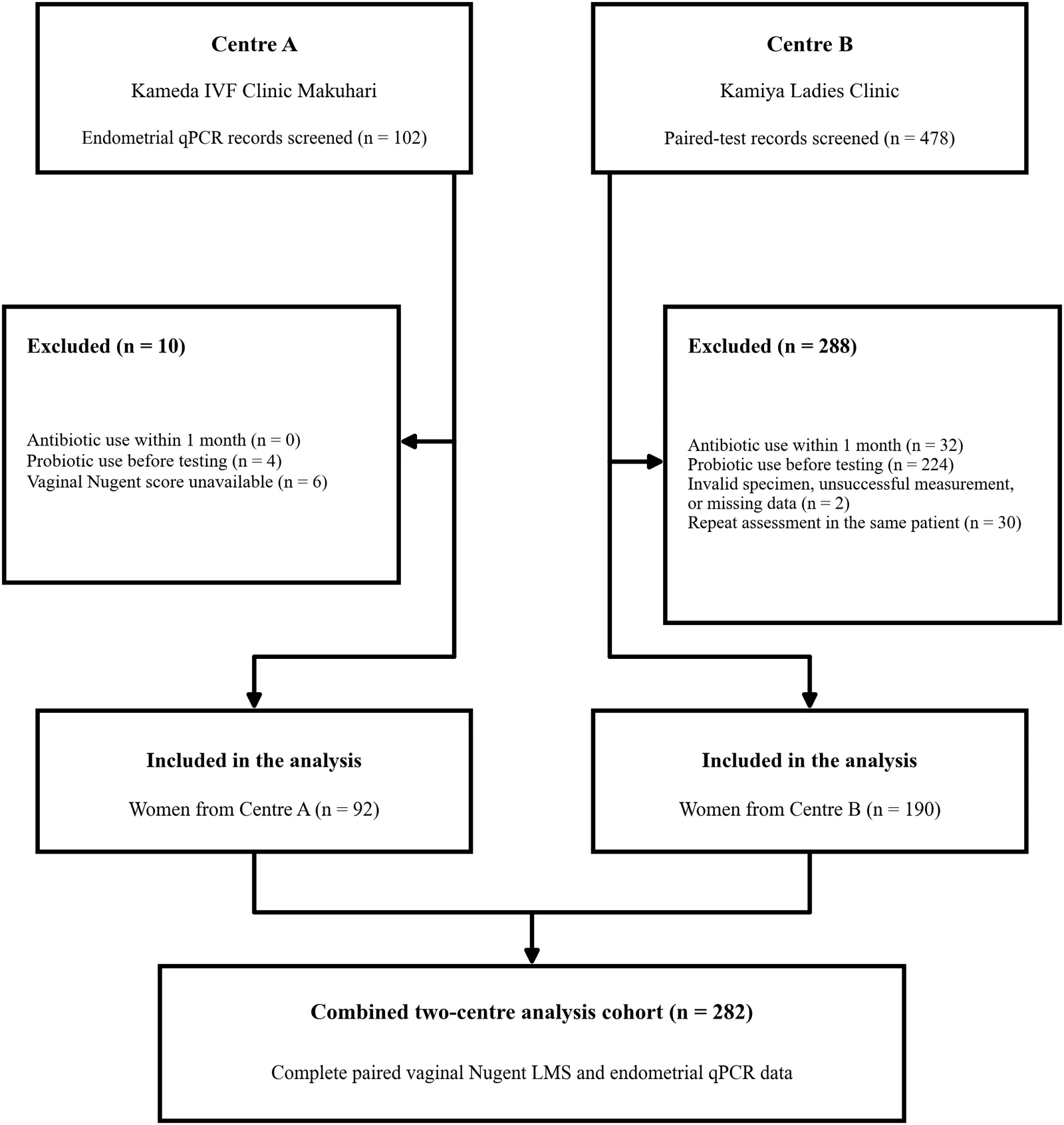
Participant recruitment flow diagram. Centre A screened 102 endometrial qPCR records and excluded 10 (antibiotic use within 1 month, n = 0; probiotic use before testing, n = 4; vaginal Nugent score unavailable, n = 6), leaving 92 women. Centre B screened 478 paired-test records and excluded 288 (antibiotic use within 1 month, n = 32; probiotic use before testing, n = 224; invalid specimen, unsuccessful measurement, or missing data, n = 2; repeat assessment in the same patient, n = 30), leaving 190 women. The final analytical cohort comprised 282 women.

### Vaginal Nugent scoring

Vaginal specimens were collected from the posterior fornix. Following Gram staining, three morphotypes were semi-quantitatively scored under oil-immersion magnification at ×1,000, according to the original Nugent method (15). The LMS (large gram-positive rods) was scored as follows: ≥30 per field = 0, 5–30 = 1, 1–4 = 2, <1 = 3, 0 = 4 (higher scores indicating fewer *Lactobacillus* morphotypes). The GMS (small gram-negative to gram-variable rods) was scored as: 0 = 0, <1 = 1, 1–4 = 2, 5–30 = 3, ≥30 = 4. The MMS (curved gram-variable rods) was scored as: 0 = 0, <5 = 1, ≥5 = 2 (as defined in the original Nugent method, scores 3–4 are undefined for MMS). The total Nugent score (LMS + GMS + MMS: 0–10) was used to classify BV status; however, LMS was analysed independently as the primary variable in this study.

### Endometrial specimen collection and qPCR analysis

Endometrial specimens were collected during the secretory phase using a sterile catheter after cervical washing and mucus removal to minimise contamination from vaginal and cervical sources. Prior to insertion, the cervix was cleaned with sterile gauze so that any residual cervical material remained on the outer surface of the catheter, while only endometrial tissue aspirated from the uterine fundus was retained within the device. When the specimen was transferred to the cryotube, the catheter was not submerged in the preservative, so that only the tissue held inside the device entered the medium, thereby minimising the introduction of cervical contaminants. DNA extracted from endometrial specimens was analysed using a customised real-time PCR panel (OpenArray platform, Thermo Fisher Scientific, Waltham, MA, USA). The panel targets 26 pathogenic species, four *Lactobacillus* species (*L. crispatus, L. gasseri, L. iners*, and *L. jensenii*), and the genus *Lactobacillus* (16). Species-specific TaqMan assays were used for amplification and detection, performed according to the manufacturer’s instructions. Analysis was conducted at a central ISO-certified and CLIA-accredited laboratory (Igenomix, Valencia, Spain). Appropriate negative controls were included throughout the analytical workflow to monitor contamination. Specimens were preserved in 1.5 mL RNA later, which stabilises nucleic acids and suppresses bacterial growth at ambient temperature, and were stored at −20 °C until processing. Within a biosafety cabinet, a tissue portion of approximately 20 mg (routinely 10–40 mg) was homogenised in 600 µL of extraction buffer, and 500 µL of the homogenate served as the analytical input; genomic copies per millilitre were referenced to this starting volume. Following the OpenArray threshold recommended by the manufacturer, a relative cycle threshold (Crt) of 28 was defined as the minimum detectable signal (one copy). The assay is semiquantitative: because the tissue portion is not weighed and varies within the 10–40 mg range, the corresponding analytical variation lies within approximately ±1 Crt cycle—within the technical limits of the assay near the limit of detection—and therefore does not materially affect the reported copy numbers. A targeted PCR assay focusing on clinically relevant species was selected, given that the endometrium is a low-biomass environment susceptible to background contamination in amplicon-based sequencing. The qPCR panel design and target species were based on the methodology reported by Moreno et al. (16). Endometrial microbiota profiles were not classified into EMMA/ALICE clinical categories (normal/abnormal); instead, *Lactobacillus* total, individual species, and pathogen copy numbers were analysed as continuous or binary (detected/undetected) variables with respect to LMS. Values below the assay detection threshold were treated as undetected (0 copies/mL). Throughout this manuscript, the term “pathogen” refers to the 26 species included in the qPCR panel as markers of dysbiosis or pathogen-associated endometrial microbiota (16). This composite includes species not classically considered pathogens (e.g., *Bifidobacterium* spp.), which are nevertheless included in the panel as markers of altered endometrial microbiota composition. Vaginal Nugent scoring and endometrial specimen collection were performed on the same day.

### Statistical analysis

Spearman rank correlation coefficients were used in the primary analysis, with robustness confirmed by partial correlation, after adjusting for centre, age, BMI, and RIF. Since undetected (below the limit of quantification) values were prevalent, both binary (detected/undetected) and quantitative analyses (detected cases only) were performed. The clinical discriminatory ability of LMS was evaluated using receiver operating characteristic (ROC) curve analysis for both endometrial pathogen detection and *L. crispatus* absence. For each LMS threshold (≥1, ≥2, ≥3, and ≥4), sensitivity, specificity, positive predictive value (PPV), and negative predictive value (NPV) were calculated with exact 95% confidence intervals. Multiple comparisons for secondary endpoints were corrected using the Benjamini–Hochberg false discovery rate (FDR) method; *L. gasseri* and *L. jensenii* were designated as exploratory. Undetected values were treated as 0 copies/mL, and robustness to threshold variation was verified using sensitivity analyses. Stratified analyses restricted to cases with GMS = 0 and MMS = 0 were performed to assess whether the association between LMS and pathogen detection persisted in the absence of elevated Gardnerella/Bacteroides or Mobiluncus morphotype scores. The “+1” in log10(copies/mL + 1) is a display constant for undetected cases. An *a priori* sample-size calculation was not performed because this retrospective study included all consecutive eligible women during the predefined study period; accordingly, the final sample size was determined by the availability of paired vaginal and endometrial assessments meeting the eligibility criteria. All analyses were performed using R version 4.3.0 (R Foundation for Statistical Computing, Vienna, Austria). p < 0.05 was considered significant. Multivariable logistic regression was used to estimate the association between LMS, modelled as an ordinal variable, and endometrial pathogen detection after adjustment for centre, age, BMI, and RIF. Results are reported as odds ratios (ORs) with 95% confidence intervals. An LMS-by-centre interaction term was examined to assess heterogeneity between centres. The 95% confidence interval for the AUC was obtained using 5,000 bootstrap resamples.

### Ethical approval

The study was approved by the Institutional Review Board of Kameda IVF Clinic Makuhari as the central ethics review board for the participating centres (IRB number: 26-002), and informed consent was obtained via the opt-out method. This study was conducted in accordance with the principles of the Declaration of Helsinki.

## Results

### Patient demographics

Patient demographics are shown in **Table 1**. The patients’ mean age was 37.0 ± 4.3 years; 35.5% had RIF (≥2 failed embryo transfers) and 25.2% had recurrent pregnancy loss (SA ≥2). Patients from Centre A showed a higher RIF rate (54.3%) than those at Centre B (26.3%), reflecting the specialty focus of Centre A on RIF.

**Table 1:** Patient demographics.

Characteristic	All (n = 282)	Centre A (n = 92)	Centre B (n = 190)
Age, years (mean ± SD)	37.0 ± 4.3	37.3 ± 4.1	36.8 ± 4.4
RIF ≥2, n (%)^†^	100 (35.5)	50 (54.3)	50 (26.3)
RPL (SA ≥2), n (%)	71 (25.2)	17 (18.5)	54 (28.4)

RIF, recurrent implantation failure; RPL, recurrent pregnancy loss; SA, spontaneous abortion.

†Centre A defined RIF as ≥2 failed embryo transfers; Centre B did not maintain a formal RIF column and applied embryo transfer count (ET) ≥2 as a working definition.

### Nugent score distribution

The distribution of Nugent score components among all patients is shown in **Table 2**. LMS 0 was the predominant category (36.5%), and LMS 4 accounted for 19.9% of cases. The GMS was 0 in 91.8% of patients, with GMS ≥1 observed in 23 cases (8.2%). The MMS was 0 in 98.9% of cases, and MMS ≥1 was observed in three cases (1.1%).

**Table 2:** Nugent score distribution (n = 282).

Score	LMS, n (%)	GMS, n (%)	MMS, n (%)
0	103 (36.5)	259 (91.8)	279 (98.9)
1	53 (18.8)	1 (0.4)	1 (0.4)
2	29 (10.3)	10 (3.5)	2 (0.7)
3	41 (14.5)	4 (1.4)	—
4	56 (19.9)	8 (2.8)	—

LMS, Lactobacillus morphotype score (0–4); GMS, Gardnerella/Bacteroides morphotype score (0–4); MMS, Mobiluncus morphotype score (0–2; scores 3–4 are not defined in the original Nugent method). —, not defined.

### LMS and endometrial pathogen detection

A significant positive correlation was observed between LMS and endometrial pathogen detection (ρ = +0.229, p < 0.001; FDR-corrected q < 0.001). Even at LMS 0, the pathogen detection rate was 23.3% (**Figure 2A**, **Supplementary Table 2**). The most frequently detected species were Bifidobacterium spp. (20.6%), G. vaginalis (13.5%), and A. vaginae (9.9%) (**Supplementary Table 3**). Stratified analysis restricted to 259 cases with GMS = 0 and MMS = 0 (91.8%) showed that the positive correlation between LMS and pathogen detection was attenuated but remained significant (ρ decreased from +0.229 to +0.164, p = 0.008; approximately 29% reduction in effect size; **Figure 2B**, **Supplementary Table 4**). The weaker correlation in this restricted subgroup indicates that concurrent GMS/MMS elevation may contribute to the overall association. In this subgroup, pathogen detection rates increased overall from 23.3% at LMS 0 to 47.7% at LMS 4.

**Figure 2:**
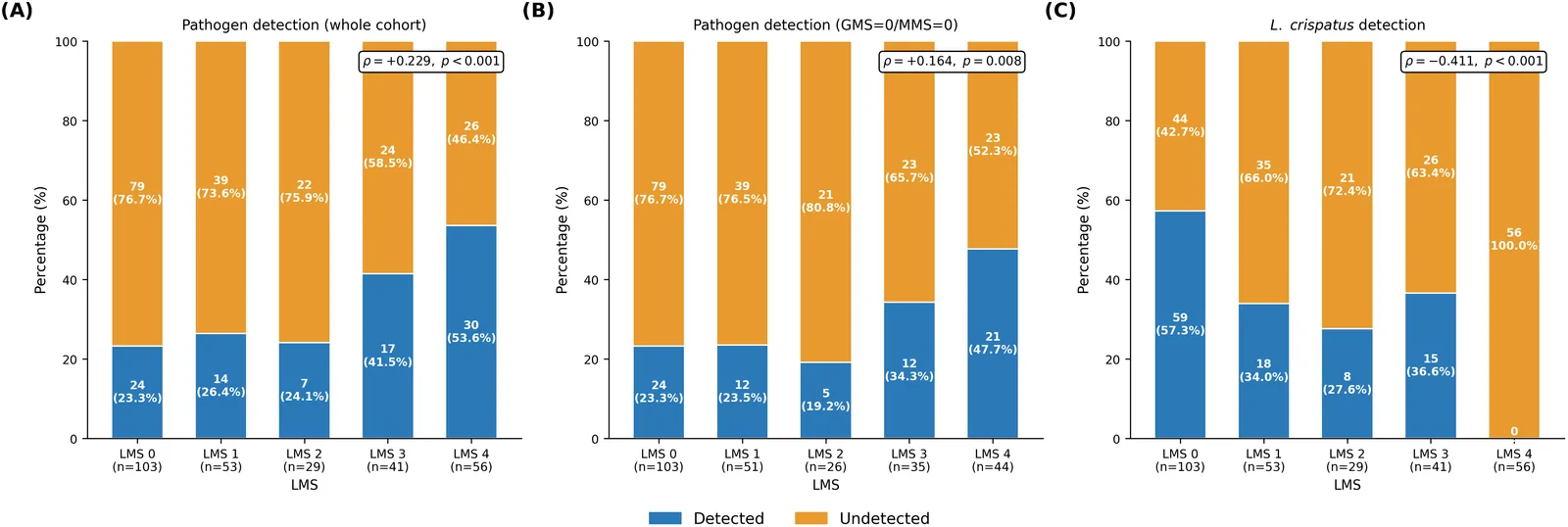
Vaginal Lactobacillus morphotype score and endometrial microbial detection. **(A)** Endometrial detection of at least one of the 26 pathogen-associated species according to LMS in the whole cohort (n = 282). Stacked bars show detected (blue) and undetected (orange) cases (Spearman ρ = 0.229; p < 0.001). **(B)** Corresponding analysis restricted to participants with Gardnerella/Bacteroides morphotype score = 0 and Mobiluncus morphotype score = 0 (n = 259), to reduce potential confounding by abnormalities in the other Nugent components (Spearman ρ = 0.164; p = 0.008). **(C)** Endometrial *L. crispatus* detection according to LMS. All 56 participants with LMS 4 were *L. crispatus*-negative (Spearman ρ = −0.411; p < 0.001). Numbers within bars indicate n (%). LMS, Lactobacillus morphotype score. Numerical data are provided in **Supplementary Table 1**, **2**, **4**.

### Multivariable and diagnostic-performance analyses

After adjustment for centre, age, BMI, and RIF, each one-point increase in LMS remained associated with higher odds of endometrial pathogen detection (adjusted OR 1.40, 95% CI 1.17–1.68; p < 0.001; **Table 3**). There was no evidence of an LMS-by-centre interaction for the primary outcome (p = 0.581). The AUC of LMS for pathogen detection was 0.636 (95% CI 0.567–0.705). Across LMS thresholds, sensitivity ranged from 32.6% to 73.9%, specificity from 41.6% to 86.3%, PPV from 38.0% to 53.6%, and NPV from 72.6% to 76.7% (**Table 4**). These values indicate limited stand-alone discrimination and do not support using LMS alone as a rule-in or rule-out test.

**Table 3:** Logistic regression analysis of LMS and endometrial pathogen detection.

Model	OR per one-point LMS increase	95% CI	p-value
Unadjusted	1.39	1.18–1.63	<0.001
Adjusted*	1.40	1.17–1.68	<0.001

*Adjusted for centre, age, BMI, and recurrent implantation failure. CI, confidence interval; LMS, *Lactobacillus* morphotype score; OR, odds ratio.

**Table 4:** Diagnostic performance of LMS thresholds for endometrial pathogen detection.

LMS-positive threshold	Sensitivity, % (95% CI)	Specificity, % (95% CI)	PPV, % (95% CI)	NPV, % (95% CI)
≥1	73.9 (63.7–82.5)	41.6 (34.5–48.9)	38.0 (30.9–45.5)	76.7 (67.3–84.5)
≥2	58.7 (47.9–68.9)	62.1 (54.8–69.0)	42.9 (34.1–52.0)	75.6 (68.1–82.1)
≥3	51.1 (40.4–61.7)	73.7 (66.8–79.8)	48.5 (38.2–58.8)	75.7 (68.8–81.7)
≥4	32.6 (23.2–43.2)	86.3 (80.6–90.9)	53.6 (39.7–67.0)	72.6 (66.3–78.3)

Endometrial pathogen detection was defined as detection of at least one of the 26 pathogen-associated species. Exact 95% confidence intervals are shown. LMS, *Lactobacillus* morphotype score; NPV, negative predictive value; PPV, positive predictive value.

### Species-specific analysis

*L. crispatus* showed the strongest negative correlation with the LMS (ρ = −0.411, p < 0.001), while *L. iners* showed a weak but significant correlation (ρ = −0.137, p = 0.021). *L. gasseri* (ρ = +0.069, p = 0.251) and *L. jensenii* (ρ = −0.084, p = 0.160) did not exhibit significant correlations with the LMS. The significance of *Lactobacillus* total (q < 0.001) and *L. crispatus* (q < 0.001) was maintained after Benjamini–Hochberg FDR correction. All 56 cases with LMS 4 (100%) were *L. crispatus*-negative (**Figure 2C**, **Supplementary Table 1**). ROC analysis revealed an AUC of 0.735 for LMS for identifying *L. crispatus* absence. At the LMS ≥4 threshold, specificity was 100.0% (95% CI 96.4–100.0) and PPV was 100.0% (95% CI 93.6–100.0), but sensitivity was only 30.8% (95% CI 24.2–38.0) and NPV was 44.2% (95% CI 37.7–51.0; **Supplementary Table 1**).

### LMS and intrauterine *Lactobacillus* total

Intrauterine *Lactobacillus* total bore a significant negative correlation with the LMS in the combined dataset (ρ = −0.488, p < 0.001). This association remained significant after partial correlation adjusting for centre, age, BMI, and RIF (partial ρ = −0.435, p < 0.001), and in both binary detection (ρ = −0.484) and quantitative analyses of detected cases only (ρ = −0.235). The total intrauterine *Lactobacillus* copy number decreased overall with an increase in the LMS, although the trend was non-monotonic at LMS 3 (**Figure 3**, **Supplementary Table 5**). The median values were 4,746, 1,482, 812, 1,676, and 0 copies/mL at LMS 0–4, respectively. The association was consistent across both centres (Centre A: ρ = −0.665; Centre B: ρ = −0.346; **Table 5**).

**Figure 3:**
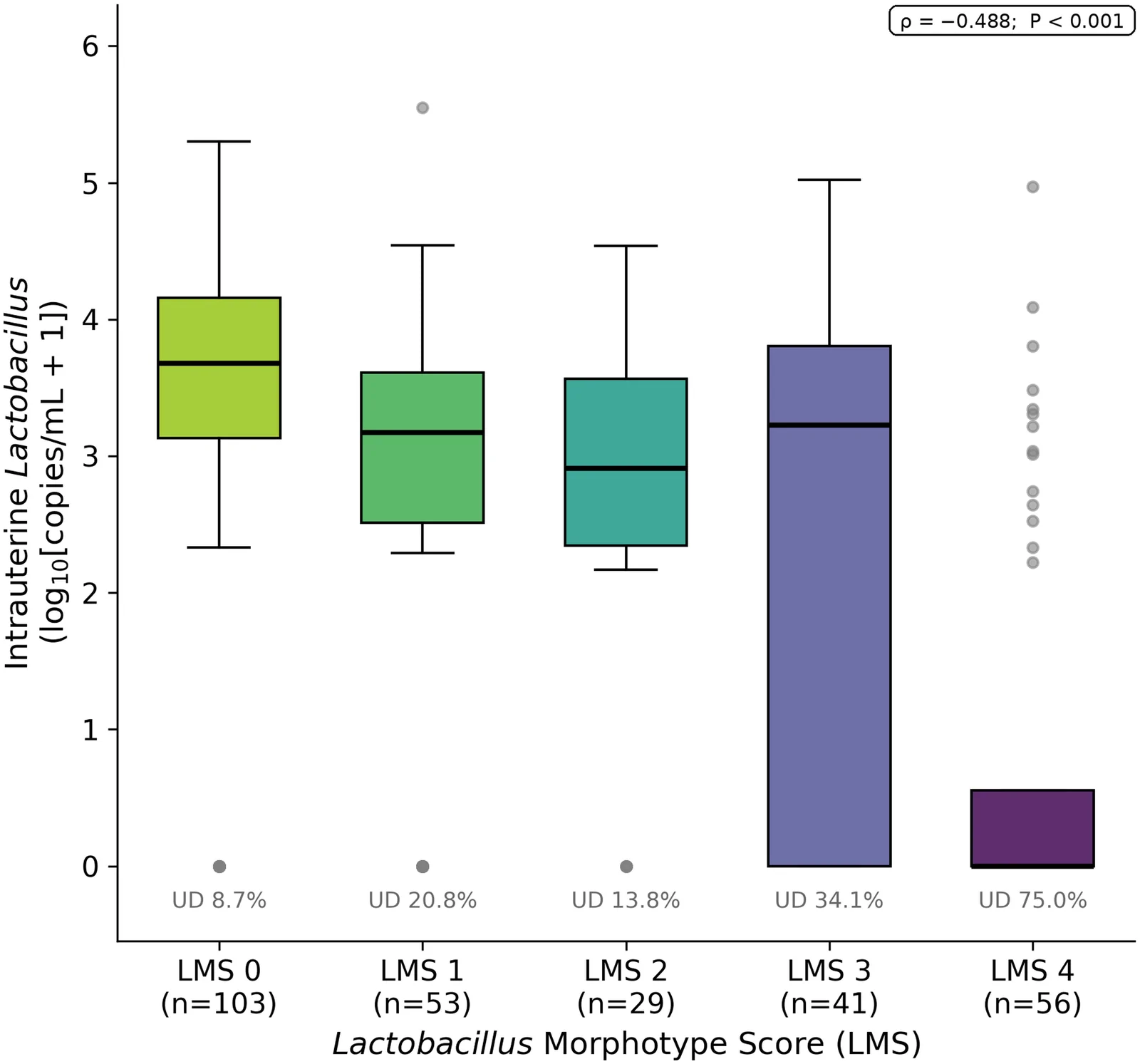
Endometrial total Lactobacillus abundance according to vaginal Lactobacillus morphotype score (LMS) (n = 282). Boxes show the median and interquartile range; whiskers and individual points are displayed according to the box-plot convention used in the analysis. The y-axis shows log_10_(copies/mL + 1), where +1 is a display constant that permits inclusion of undetected values. UD indicates the percentage of undetected samples in each LMS category (Spearman ρ = −0.488; p < 0.001). Numerical data are provided in **Supplementary Table 5**.

**Table 5:** Inter-centre consistency: Spearman correlations between the LMS and endometrial microbiota variables.

Variable	All ρ (p)	Centre A ρ (p)	Centre B ρ (p)
Lactobacillus total	−0.488 (<0.001***)	−0.665 (<0.001***)	−0.346 (<0.001***)
L. crispatus	−0.411 (<0.001***)	−0.435 (<0.001***)	−0.285 (<0.001***)
L. iners	−0.137 (0.021*)	−0.308 (0.003**)	−0.124 (0.087)
L. gasseri	+0.069 (0.251)	−0.163 (0.119)	+0.152 (0.036*)
L. jensenii	−0.084 (0.160)	−0.129 (0.222)	−0.073 (0.316)
Pathogens (26 species)	+0.229 (<0.001***)	+0.293 (0.005**)	+0.167 (0.022*)

ρ, Spearman rank correlation coefficient. *p < 0.05; **p < 0.01; ***p < 0.001. Centre A: Kameda IVF Clinic Makuhari (n = 92); Centre B: Kamiya Ladies Clinic (n = 190). LMS, Lactobacillus morphotype score.

### Inter-centre consistency

Inter-centre consistency of the Spearman correlations is shown in **Table 5**. *Lactobacillus* total, *L. crispatus*, and pathogens (26 species) were significantly correlated at both centres, with consistent directions (pathogen detection: Centre A, ρ = +0.293, p = 0.005; Centre B, ρ = +0.167, p = 0.022). *L. iners* was significant at Centre A (ρ = −0.308, p < 0.01) but not at Centre B (ρ = −0.124, p = 0.087). *L. gasseri* showed a reverse correlation direction between facilities, and *L. jensenii* was not significant at either facility.

## Discussion

This study examined whether vaginal Nugent LMS was associated with endometrial pathogen detection and intrauterine *Lactobacillus* abundance in women with infertility. LMS showed a statistically significant but weak association with pathogen detection (ρ = +0.229), whereas its inverse association with total intrauterine *Lactobacillus* abundance was stronger (ρ = −0.488). The adjusted logistic model confirmed that the pathogen association persisted after accounting for centre, age, BMI, and RIF; nevertheless, the AUC of 0.636 and the threshold-specific predictive values indicated limited discrimination. Taken together, the findings show group-level concordance between vaginal and endometrial *Lactobacillus* depletion but do not establish surrogate or substitute validity for LMS.

*L. crispatus* showed the strongest correlation (ρ = −0.411), and at LMS 4, all cases (100%) were intrauterine *L. crispatus*-negative. *L. iners* showed a weaker correlation (ρ = −0.137). Verstraelen et al. reported that *L. crispatus*-dominant vaginal microbiota is highly stable, whereas *L. iners*-dominant microbiota is prone to dysbiosis transition (17). *L. iners* may be counted as a *Lactobacillus* morphotype under Gram staining, but its morphology is more variable compared with *L. crispatus*, suggesting differential LMS sensitivity between species (18). The selective reflection of *L. crispatus* by LMS is consistent with this morphological difference. Oguri et al. (19) reported favourable pregnancy outcomes in women with dominant vaginal *L. crispatus* in early pregnancy in a Japanese maternal–neonatal cohort, underscoring the clinical importance of *L. crispatus* in reproductive medicine. Kadogami et al. (20) reported the lowest implantation rates with dominant intrauterine *L. iners*, consistent with our finding that the LMS selectively reflects *L. crispatus* with a weaker correlation with *L. iners*. The *L. crispatus* detection rate also showed a non-monotonic pattern (57.3%, 34.0%, 27.6%, 36.6%, and 0% from LMS 0 to LMS 4, respectively). The transient elevation at LMS 3 likely reflects the heterogeneity of this transitional Nugent category—by definition, LMS 3 corresponds to <1 *Lactobacillus* morphotype per field, encompassing both women with markedly reduced but residual *L. crispatus* colonisation and women approaching complete *Lactobacillus* depletion. This heterogeneity is also reflected in the wide interquartile range of intrauterine *Lactobacillus* at LMS 3 (0–6,376 copies/mL). In contrast, LMS 4 (no *Lactobacillus* morphotypes detected) corresponded to a uniformly *L. crispatus*-negative endometrial profile (specificity 1.000), indicating a highly specific but poorly sensitive association between LMS 4 and *L. crispatus* absence in this cohort. Thus, LMS 4 identified only a subset of *L. crispatus*-negative cases and should not be interpreted as a stand-alone test.

Several lines of biological interpretation merit careful consideration. First, qPCR-based DNA quantification cannot distinguish between viable bacteria, non-viable bacteria and residual free DNA. Sola-Leyva et al. (21) reported that, in paired endometrial biopsies, *Lactobacillus* was detectable by DNA analysis but showed no transcriptional activity by RNA analysis. The endometrial cavity is also less hospitable to active *Lactobacillus* colonisation than the vagina, given its near-neutral pH (22, 23).

A second consideration is carry-over contamination during sampling. Although this was minimised by cervical washing and by a transfer step in which the catheter was not submerged in the preservative, so that only tissue retained inside the device entered the medium, residual carry-over from the lower genital tract cannot be fully excluded (24). Women with a high LMS—and therefore reduced vaginal *Lactobacillus*—might show reduced endometrial *Lactobacillus* DNA simply because less *Lactobacillus* is available for inadvertent transfer during catheter passage. The LMS–intrauterine *Lactobacillus* correlation could therefore partly reflect a sampling artefact rather than a biological linkage.

The analysis restricted to GMS = 0 and MMS = 0 reduced the influence of visible Gardnerella/Bacteroides and Mobiluncus morphotype abnormalities, and the LMS–pathogen association remained statistically significant (ρ = +0.164). However, this restriction does not address carry-over during transcervical sampling. Prior paired DNA/RNA work provides relevant biological context but does not resolve this source of bias in the present cohort (21). Accordingly, we cannot determine whether the observed concordance reflects true biological continuity, transfer of lower-tract microorganisms during sampling, or both.

Accordingly, the present results should be interpreted as associations between vaginal LMS and selected endometrial qPCR findings. They do not establish that LMS is a surrogate for the endometrial microbiota, nor do they demonstrate that use of LMS to select patients for endometrial testing improves clinical outcomes.

Regarding the association with pathogens, LMS was positively correlated with endometrial pathogen detection (ρ = +0.229). Since the Nugent LMS evaluates only *Lactobacillus* morphotype and does not directly assess pathogen morphology, this correlation may reflect an underlying biological relationship, transfer of lower-tract microorganisms during sampling, or both. However, the pathogen detection rate at LMS 0 was 23.3%, indicating that a low LMS does not guarantee pathogen absence. In the GMS = 0/MMS = 0 stratum, the LMS–pathogen correlation was attenuated (ρ from +0.229 to +0.164) but remained significant (p = 0.008). This pattern is consistent with partial confounding by concurrent GMS/MMS elevation, while supporting an independent component of the LMS–pathogen association. Pathogen detection was 78.3% in GMS ≥1 cases, but 28.6% even with GMS = 0, highlighting limitations in using individual Nugent components to assess endometrial pathogens. Stratified analysis of major pathogens (Bifidobacterium spp., G. vaginalis, and A. vaginae) showed an overall increase in detection rates with rising LMS (**Supplementary Table 6**). As previously noted, dysbiosis-related pathogens may maintain metabolic activity even in the endometrial environment, and qPCR-based pathogen detection represents a molecular finding whose clinical significance requires outcome-based validation (21). The weak correlation, AUC of 0.636, and threshold-specific predictive values indicate substantial overlap in endometrial pathogen status across LMS categories. In particular, the 23.3% pathogen detection rate at LMS 0 corresponds to an NPV of only 76.7% when LMS ≥1 is treated as a positive test. Therefore, LMS 0 cannot be used to exclude endometrial pathogen detection. PPV and NPV are also prevalence-dependent and may differ in other populations.

Regarding clinical positioning, the present study does not establish a specific protocol or LMS threshold at which endometrial microbiota testing should be performed. LMS should not independently trigger endometrial testing or antimicrobial treatment. At most, an elevated LMS may provide supplementary contextual information in selected patients in whom endometrial evaluation is already being considered for other clinical reasons. Prospective studies are required to determine whether LMS-based stratification reduces unnecessary testing, alters treatment decisions, or improves reproductive outcomes.

Importantly, vaginal LMS cannot substitute for direct endometrial microbiota assessment, and a low LMS cannot rule out endometrial pathogen detection. Conversely, the present findings do not establish that direct microbiota testing is routinely indicated or clinically beneficial. LMS should therefore be regarded as a preliminary non-invasive indicator whose potential clinical role requires prospective validation rather than as a validated screening or diagnostic test.

Because definitions of endometrial dysbiosis are not standardised, the observed group-level associations cannot be translated into an individual microbiota classification. LMS should not be used to classify endometrial microbiota status.

The limitations of this study include the inability to completely exclude contamination from vaginal/cervical sources during endometrial specimen collection (carry-over contamination) (24). In endometrial microbiota research, the type of specimen, degree of vaginal/cervical contact during collection, and presence of a protective sheath affect contamination risk and contribute to inter-study inconsistency (7). Furthermore, no assisted reproduction or pregnancy outcomes—such as implantation, clinical pregnancy, or live birth—were evaluated; the present findings therefore cannot establish that the LMS predicts reproductive prognosis, and its clinical value remains to be determined in outcome-based prospective studies. In addition, despite the consistent direction and significance of correlations, the magnitude differed between facilities (total *Lactobacillus*: ρ = −0.665 at Centre A vs. −0.346 at Centre B). This heterogeneity likely reflects several factors. First, Centre A had a markedly higher proportion of patients with RIF (54.3% vs. 26.3% at Centre B), who more frequently exhibit endometrial *Lactobacillus* depletion, which may amplify the LMS–microbiota correlation in this enriched population. Second, although both centres applied the same Nugent scoring criteria and centrally analysed all endometrial samples at Igenomix, slide reading was performed by independent laboratory technicians at each site, and formal inter-rater reliability could not be assessed owing to the retrospective design. Despite this between-site variation in effect magnitude, the direction of the primary correlation was preserved at both centres (Centre A, p = 0.005; Centre B, p = 0.022), and no significant LMS-by-centre interaction was detected for pathogen detection (p = 0.581), supporting the reproducibility of the central finding. The retrospective design also introduced the possibility of selection bias, and future prospective studies are warranted.

Strengths include the two-centre design, consistency after adjustment for centre, age, BMI, and RIF, sensitivity analyses across qPCR detection thresholds, standardised Nugent scoring, and qPCR-based copy-number assessment. Generalisability is limited because endometrial samples were obtained only in the secretory phase and participants were Japanese women attending specialised fertility centres. The study did not evaluate reproductive outcomes and cannot establish prognostic value. Prospective multicentre studies should incorporate protected sampling with paired contamination controls, prespecified LMS thresholds, external validation, and reproductive outcomes.

## Conclusions

Higher vaginal LMS was associated with lower intrauterine *Lactobacillus* abundance and, more weakly, with endometrial pathogen detection in Japanese women with infertility. However, limited discriminatory performance and the occurrence of pathogen detection among women with LMS 0 indicate that LMS cannot substitute for direct endometrial assessment or reliably exclude endometrial microbial abnormalities. LMS should therefore be regarded as a preliminary non-invasive indicator rather than a validated screening or diagnostic test. Prospective external validation incorporating reproductive outcomes is required before clinical implementation.

## Data Availability

The raw data supporting the conclusions of this article will be made available by the authors, without undue reservation.
