# Supplementary material for: Vaginal Nugent Lactobacillus morphotype score and endometrial pathogen detection: a two-centre retrospective study
Source: Front Endocrinol (Lausanne). 2026 Sep 14;17:1926579. doi: 10.3389/fendo.2026.1926579 (PMC13616690; doi:10.3389/fendo.2026.1926579)
Supplement: Supplementary file 1 [file DataSheet1.docx]

**Supplementary Table S1. *Lactobacillus crispatus* detection by LMS**

| **LMS** | **n** | **Detected, n (%)** | **Undetected, n (%)** |
| --- | --- | --- | --- |
| 0 | 103 | 59 (57.3) | 44 (42.7) |
| 1 | 53 | 18 (34.0) | 35 (66.0) |
| 2 | 29 | 8 (27.6) | 21 (72.4) |
| 3 | 41 | 15 (36.6) | 26 (63.4) |
| 4 | 56 | 0 (0.0) | 56 (100.0) |

*Detected was defined as an L. crispatus copy number > 0. ROC analysis: AUC for L. crispatus absence = 0.735. At the LMS ≥ 4 threshold, sensitivity was 30.8% (95% CI 24.2–38.0), specificity was 100.0% (95% CI 96.4–100.0), PPV was 100.0% (95% CI 93.6–100.0), and NPV was 44.2% (95% CI 37.7–51.0). The target condition was absence of endometrial L. crispatus, which was absent in 182 of 282 women (64.5%). Positive and negative predictive values depend on this prevalence and will differ in populations with a different underlying rate. Spearman ρ = −0.411, p < 0.001. AUC, area under the curve; CI, confidence interval; LMS, Lactobacillus morphotype score; NPV, negative predictive value; PPV, positive predictive value; ROC, receiver operating characteristic. Numerical data for Figure 2C.*

**Supplementary Table S2. Endometrial pathogen detection rate by LMS (whole cohort)**

| **LMS** | **n** | **Pathogen detected, n (%)** |
| --- | --- | --- |
| 0 | 103 | 24 (23.3) |
| 1 | 53 | 14 (26.4) |
| 2 | 29 | 7 (24.1) |
| 3 | 41 | 17 (41.5) |
| 4 | 56 | 30 (53.6) |

*Pathogen detected, at least one of the 26 pathogen-associated species detected (copy number > 0). Spearman ρ = +0.229, p < 0.001. LMS, Lactobacillus morphotype score. Numerical data for Figure 2A.*

**Supplementary Table S3. Pathogen-associated species detected in the entire cohort (n = 282)**

| **Pathogen-associated species** | **n detected** | **Detection rate (%)** |
| --- | --- | --- |
| *Bifidobacterium* spp. | 58 | 20.6 |
| *Gardnerella vaginalis* | 38 | 13.5 |
| *Atopobium vaginae* | 28 | 9.9 |
| *Prevotella bivia* | 10 | 3.5 |
| *Mobiluncus* spp. | 8 | 2.8 |
| *Prevotella disiens* | 4 | 1.4 |
| *Sneathia* spp. | 4 | 1.4 |
| *Escherichia coli* | 4 | 1.4 |
| *Streptococcus agalactiae/S. viridans* | 4 | 1.4 |
| *Ureaplasma urealyticum* | 3 | 1.1 |
| *Peptostreptococcus anaerobius* | 2 | 0.7 |
| *Enterococcus faecalis* | 2 | 0.7 |
| Others* | 3 | — |

**Others: Bacteroides fragilis, Chlamydia trachomatis, Staphylococcus aureus (1 case each). Detection rate calculated with n = 282.*

**Supplementary Table S4. Endometrial pathogen detection rate by LMS within the GMS = 0 / MMS = 0 stratum**

| **LMS** | **n** | **Pathogen detected, n (%)** |
| --- | --- | --- |
| 0 | 103 | 24 (23.3) |
| 1 | 51 | 12 (23.5) |
| 2 | 26 | 5 (19.2) |
| 3 | 35 | 12 (34.3) |
| 4 | 44 | 21 (47.7) |

*Analysis restricted to 259 cases (91.8%) with Gardnerella/Bacteroides morphotype score (GMS) = 0 and Mobiluncus morphotype score (MMS) = 0 to reduce potential confounding by abnormalities in the other Nugent components. Spearman ρ = +0.164, p = 0.008. LMS, Lactobacillus morphotype score. Numerical data for Figure 2B.*

**Supplementary Table S5. Intrauterine *Lactobacillus* total by LMS**

| **LMS** | **n** | **Median** | **IQR** | **Undetected, n (%)** |
| --- | --- | --- | --- | --- |
| 0 | 103 | 4,746 | 1,351–14,364 | 9 (8.7) |
| 1 | 53 | 1,482 | 324–4,052 | 11 (20.8) |
| 2 | 29 | 812 | 220–3,662 | 4 (13.8) |
| 3 | 41 | 1,676 | 0–6,376 | 14 (34.1) |
| 4 | 56 | 0 | 0–42 | 42 (75.0) |

*Median and IQR are in copies/mL. Undetected, copy number below the lower limit of quantification (treated as 0 copies/mL). Spearman ρ = −0.488, p < 0.001. IQR, interquartile range; LMS, Lactobacillus morphotype score. Numerical data for Figure 3.*

**Supplementary Table S6. Major pathogen-associated species detection rate by LMS**

| **Pathogen-associated species** | **LMS 0 (n = 103)** | **LMS 1 (n = 53)** | **LMS 2 (n = 29)** | **LMS 3 (n = 41)** | **LMS 4 (n = 56)** |
| --- | --- | --- | --- | --- | --- |
| *Bifidobacterium* spp. | 15.5% | 18.9% | 13.8% | 29.3% | 28.6% |
| *Gardnerella vaginalis* | 6.8% | 11.3% | 13.8% | 22.0% | 21.4% |
| *Atopobium vaginae* | 1.9% | 5.7% | 13.8% | 12.2% | 25.0% |

*Detection rate (%) = proportion of cases with copy number > 0 within each LMS group. LMS, Lactobacillus morphotype score.*
